# Diversity, Function and Regulation of Cell Surface and Intracellular Immune Receptors in *Solanaceae*

**DOI:** 10.3390/plants9040434

**Published:** 2020-04-01

**Authors:** Jong Hum Kim, Christian Danve M. Castroverde

**Affiliations:** 1 Department of Energy Plant Research Laboratory, Michigan State University, East Lansing, MI 48824, USA; 2 Howard Hughes Medical Institute, Michigan State University, East Lansing, MI 48824, USA; 3 Department of Biology, Wilfrid Laurier University, Waterloo, ON N2L 3C5, Canada

**Keywords:** plant immunity, solanaceous plants, pattern recognition receptor, NB-LRR receptor, resistance protein, immune receptor, host–pathogen interaction

## Abstract

The first layer of the plant immune system comprises plasma membrane-localized receptor proteins and intracellular receptors of the nucleotide-binding leucine-rich repeat protein superfamily. Together, these immune receptors act as a network of surveillance machines in recognizing extracellular and intracellular pathogen invasion-derived molecules, ranging from conserved structural epitopes to virulence-promoting effectors. Successful pathogen recognition leads to physiological and molecular changes in the host plants, which are critical for counteracting and defending against biotic attack. A breadth of significant insights and conceptual advances have been derived from decades of research in various model plant species regarding the structural complexity, functional diversity, and regulatory mechanisms of these plant immune receptors. In this article, we review the current state-of-the-art of how these host surveillance proteins function and how they are regulated. We will focus on the latest progress made in plant species belonging to the *Solanaceae* family, because of their tremendous importance as model organisms and agriculturally valuable crops.

## 1. Introduction

*Solanaceae*, a family of flowering dicot plants including valuable crops such as tomato, potato, and pepper, have been studied for a long time because of their agricultural and economic importance. Because these plants are targeted by various pathogens, the insights on plant–pathogen interactions are important to maintain these plants’ agronomic value [1]. Research for the past several decades have demonstrated that pathogens and plants have been co-evolving through continuous pressure for survival. Pathogens have effective and versatile weapons to conquer their host, while hosts also have diverse shields and sensors to avoid and detect invasions by pathogens [2]. 

As a first layer of the plant innate immune system, receptors have been adopted and developed to sense invaders. In the apoplast where the plant plasma membrane is the first venue encountered by pathogen invasion, it proves effective for plants to localize diverse receptors at this front line. These receptors usually detect highly conserved molecules, such as small peptides and carbohydrates, found in a group of microorganisms. These invasion patterns or ligands are typically microbe- or pathogen-associated molecular patterns (MAMPs/PAMPs) and the resulting immune response is called pattern-triggered immunity (PTI). Downstream PTI responses include production of reactive oxygen species, alterations in plant cell wall, and induction of antimicrobial compounds, which are delicately mediated by complex signal transduction pathways [3].

In contrast to pattern-triggered immunity, host resistance (R) proteins of the nucleotide-binding leucine-rich receptor (NLR) family are specialized intracellular receptors for gene-for-gene immunity. These NLRs target matching avirulence (Avr) factors in a pathogen [2]. To enhance their fitness in plant tissues, pathogens have adopted molecular tools which are secreted to the extracellular matrix or into the plant cell. Since effector proteins from Avr genes secreted into the plant cell can suppress the plant immune system, host receptors for these foreign molecules are usually located in the intracellular space [2]. Because these Avr factors tend to be virulence effectors that can shut down certain aspects of plant innate immunity, this NLR-activated response is collectively termed effector-triggered immunity (ETI) [2].

Plant–pathogen interactions occur under dynamically changing environmental conditions, which can modulate plant defense responses in several cases [4]. Therefore, plants have developed diverse mechanisms to find balance and maximize fitness between plant–pathogen interactions and environmental conditions.

In this review, we focus on immune receptors in *Solanaceae*, especially how they recognize their respective ligands, transduce the signal downstream, and are regulated by external factors.

## 2. Cell Surface Immune Receptors in Solanaceous Plants

### 2.1. Structure

Cell surface immune receptors are typically leucine-rich repeat transmembrane proteins, with their (1) extracellular side responsible for binding and recognizing the ligand or invasion pattern, (2) a transmembrane domain responsible for properly tethering the protein within the plasma membrane, and (3) a cytoplasmic side for downstream signaling. Surface immune receptors can be classified as either receptor-like kinases (RLKs) or receptor-like proteins (RLPs), depending on the presence or absence of kinases activity in their cytoplasmic tail (for extensive review, see Albert et al) [5].

The extracellular domain is used for the perception and binding of molecular patterns that signal impending invasion or danger. As shown in Table 1, the origin and biochemical nature of these invasion patterns are diverse. They could be derived from the invading organism termed microbe- or pathogen-associated molecular patterns or MAMPs/PAMPs, or they could be derived from the host itself due to release upon colonization or damage termed damage-associated molecular patterns (DAMPs) [3]. These ligands could be proteinaceous, as in the case of pathogen-derived flagellin epitopes (flg22 and flgII-28) or host-derived systemin [6,7,8]. They could be carbohydrate-based or lipid-based, as in the case of pathogen-derived chitin and lipopolysaccharide [9,10]. Finally, although they have not been demonstrated in solanaceous plants yet, these ligands could also be nucleotide-based, as in the case of pathogen-derived RNAs or host-derived ATP [11,12]. 

### 2.2. Ligand Recognition and Signaling

Extracellular danger signals or invasion patterns are perceived by supramolecular protein complexes at the plasma membrane, consisting of: (1) The primary ligand-binding immune receptor protein, (2) one or two co-receptors, (3) cytoplasmic kinases, and (4) regulatory proteins. A comprehensive list of solanaceous cell surface immune RLPs and RLKs are outlined in Table 1.

These immune receptors have been shown to either homodimerize or heterodimerize. Homodimerization has been demonstrated during the perception of chitin by Arabidopsis CERK1, which is a member of the LysM family of RLKs [13]. One of the tomato CERK1 homologs is SlLYK1, which is involved in chitin-induced responses [9]. Two other tomato CERK1 homologs Bti9 and SlLyk13 are known to be targets of the bacterial virulence effector AvrPtoB and play a role in plant immunity [14]. Whether SlLYK1, Bti9, and SlLyk13 participate in direct recognition of a certain pathogen-derived ligand through homodimerization has yet to be demonstrated.

Heterodimerization has been shown in several solanaceous cell surface immune receptors. RLKs, like FLS2 (flagellin sensing 2), FLS3 (flagellin sensing 3), and CORE (cold shock protein receptor) tend to form heterodimers with the co-receptor BAK1 after ligand binding [6,15,16]. On the other hand, because RLPs lack a cytoplasmic kinase domain for downstream immune signaling, they tend to be in a constitutive complex with tomato adaptor kinase SOBIR1 (Suppressor of BIR1-1) [17]. This constitutive immune RLP-adaptor kinase bimolecular complex serves as the functional equivalent of immune RLKs [18]. After ligand binding, the RLP-SOBIR1 complex interacts with BAK1, as shown in various examples like NbCSPR (Receptor-like protein required for csp22 responsiveness) in *Nicotiana benthamiana*, Cf, Ve, and EIX in tomato, and ELR in potato [17,19,20]. Because the ligand for CuRe1 (Cuscuta Receptor 1) has not yet been identified, its interaction with BAK1 has yet to be demonstrated [21].

BAK1 and related co-receptor kinases play a central role not just in plant immunity but in plant growth and development as well [15]. As in the case of FLS2-BAK1, the binding of its ligand flg22 stabilizes the supramolecular protein complex by acting like a molecular adhesive [22]. BAK1 also serves to strengthen and potentiate the phosphorylation events in the immune receptor protein [15]. Solanaceous plant orthologs of BAK1 have been identified. In tomato, SlSERK3A and SlSERK3B can partially rescue the Arabidopsis *bak1* mutant phenotype [23]. In potato, StSERK3A/B mediate defense responses induced by the DAMP Pep-13 [24].

The transmembrane immune receptor-co-receptor complex transduces signals through receptor-like cytoplasmic kinases (RLCKs). One well-characterized RLCK in Arabidopsis is BIK1, for which homologs in solanaceous plants occur. BIK1 is part of the large multigenic RLCK-VII subfamily and participates in the phosphorylation amplification cascade [25]. It is released upon ligand binding from its interaction with the receptor-co-receptor complex [25]. A tomato RLCK called tomato protein kinase 1b (TPK1b) is the BIK1 ortholog as it functionally complements the Arabidopsis *bik1* mutant [26]. TPK1b regulates defense responses to necrotrophic pathogens and insects, and has been shown to interact with the PEPR1/2 ORTHOLOG RECEPTOR-LIKE KINASE1 (PORK1) [26]. Although the TPK1b-PORK1 modulates gene expression in response to the wounding hormone systemin, it remains to be seen whether it directly binds the systemin receptor SYR1 or if its effect is due to more indirect means [26]. Also, the tomato Pti1a and Pti1b proteins act as RLCKs transducing the immune signals by the invasion pattern flg22 [27]. In pepper, the RLCK CaPIK1 has been shown to be involved in plant defense responses and cell death, but whether it directly interacts with surface receptors has yet to be demonstrated. Deciphering the various molecular substrates of diverse RLCKs are key to identifying the downstream components of RLP/RLK-mediated immune signaling [28].

Signal transduction during cell surface receptor-mediated immunity can be achieved through phosphorylation cascades of MAP kinases and/or calcium-dependent protein kinases (CDPKs). In tomato, genome-wide analyses identified 89 MAPKKK, 5 MAPKK and 13 MAPK genes [29,30]. Also in tomato, genome-wide analyses identified 29 CDPKs and 6 CDPK-related kinases (CRKs), with some involved in basal disease resistance [31]. However, which of these MAPKs and CDPKs directly participate in immune signal transduction have yet to be biochemically demonstrated. Detailed functional and high-throughput analyses in Arabidopsis have shown that these MAPKs and CDPKs could directly phosphorylate transcription factors that translocate to the nucleus to directly regulate immune gene expression [32,33]. Elucidating the various downstream molecular targets of MAPKs and CDPKs in solanaceous plants will broaden our understanding of innate immune signaling and downstream defense gene regulation. 

### 2.3. Expression and Regulation

Solanaceous cell surface immune receptors are regulated at multiple levels: (1) Transcriptional and epigenetic, (2) post-transcriptional, and (3) post-translational regulatory mechanisms.

At the transcriptional level, tomato immune receptors have been found to be induced by ergosterol and squalene from the fungal symbiont *Trichoderma* [34]. In solanaceous plants, examples of RLP/RLK and NLR genes can be induced by wounding [35,36], hormone treatments [10,35,37], pathogen infection [10,37,38,39,40] and effector gene expression [37,41]. In Arabidopsis, both RLP and NLR genes can be induced by a range of environmental stresses and different hormones [42,43].

Epigenetic regulation has been shown to occur in Arabidopsis. Known immune receptor genes (both cell surface and intracellular) can be affected by epigenetic changes in proximal and distal transposons through trans-regulatory small RNAs dependent on RNA-directed DNA methylation (RdDM) [44]. This is an interesting observation since the tomato *Ve1* RLP gene promoter has been shown to possess differential transcriptional activities in its native chromatin context and when it is introduced elsewhere in the genome [37]. 

Post-transcriptional regulation of cell surface immune receptor mRNAs can occur in various ways, including alternative splicing. In un-induced/uninfected tobacco cells, *Nt-Sd-RLK* is produced as a shorter transcript by alternative splicing, where only the extracellular domain is encoded by the mRNA [45]. When the invasion pattern LPS is used to induce the cells, a longer transcript is produced containing the cytoplasmic kinase domain [45]. Because the kinase domain is associated with downstream signaling, this presumably modulates defense responses and maintains immune homeostasis [45].

Finally, post-translational mechanisms of regulation include different processes like differential protein modifications, protein degradation, protein stabilization, protein interaction, and protein trafficking. These regulatory mechanisms allow for proper immune homeostasis and defense signaling in a controlled manner.

In the tomato immune receptor-Pti1b RLCK module [46], a phosphatase termed pattern-triggered immunity inhibiting PP2C 1 (Pic1) negatively controls Pti1b autophosphorylation and activity, which relays flagellin-induced immune signaling [46]. Pti1b autophosphorylation on threonine-233 is abolished when Pic1 is present [46]. An arginine-to-cysteine substitution on residue 240 made Pti1b constitutively active and resistant to Pic1 dephosphorylation, although the Pti1b-Pic1 interaction remained intact [46]. Apart from phosphorylation, immune receptor SUMOylation can modulate its function and downstream immune responses, as in the case of Arabidopsis FLS2 [47], but it has yet to be shown biochemically in solanaceous plants.

Regulation by protein degradation has been demonstrated in the flg22-FLS2-SlPUB13 ubiquitination complex [48]. The tomato homolog of the U-box type E3 ligase PUB13 works with group III E2 enzymes for FLS2 ubiquitination and eventual degradation [48].

Apart from protein degradation, protein stabilization is also key to proper functioning of cell surface immune receptors. Molecular scaffolds, like tomato TFT1 (a 14-3-3) protein, is important in immunity against *Xanthomonas* and is a target of the *Xanthomonas* virulence effector XopN [49]. The multifunctional cochaperone Hsp70/90 organizing protein Hop/Sti1 is important for signaling and response to chitin [50]. 

Differential protein interaction positively or negatively modulates plant immunity. Tomato immune receptor-mediated responses can be negatively regulated by SlBIR3, which interacts with BAK1 [51]. This constitutive BIR3-BAK1 interaction is relieved upon ligand binding, thereby freeing the BAK1 co-receptor to associate with the immune receptor complex [51].

Finally, protein trafficking plays a great role in regulating immune receptor function. SlPRA1A interacts with the tomato EIX receptor and reduces protein levels. In addition, SlPRA1A is presumed to redirect EIX from endosome to the vacuole for degradation [52]. SlPRA1A influences levels of other immune RLPs but not RLKs, suggesting a bifurcation in how these two sets of cell surface receptors are trafficked intracellularly [52]. Endocytosis is regulated by certain protein-protein interaction modules, including those containing the Eps15 homology domain (EHD). In tomato, the EHD2 directly interacts with EIX2 and mediates its internalization and downstream defense outputs [53]. This is specific to RLP as it did not affect the RLK FLS2 (Bar and Avni, 2009). Endocytosis after ligand binding is necessary to replenish new ligand-free receptors, which is known to be mediated by SCD1 and ESCRT1 in Arabidopsis [54,55]. The identification and characterization of solanaceous orthologs of this trafficking components will pave the way for expanding the principles of plant immune signaling and also to relating its crosstalk with other physiological processes. 

## 3. Intracellular Immune Receptors in Solanaceous Plants

### 3.1. Structure

Intracellular immune receptors that have been studied in Solanaceous plants so far are summarized in Table 2. Although various intracellular immune receptors that bind different ligands from diverse pathogens have been reported, they could be classified by their conserved domains [85]. The nucleotide binding domain (NB-ARC; nucleotide-binding adaptor shared by APAF-1, R proteins, and CED-4) and leucine-rich repeat (LRR) are located in the central and C-terminal regions of these immune receptors [85]. These NB-LRR receptors or NLRs could be divided into TNL (TIR-NB-LRR; Toll/interleukin-1 receptor-nucleotide-binding-leucine-rich repeat) and CNL (CC-NB-LRR; coiled coil-nucleotide-binding-leucine-rich repeat) subgroups by the presence of additional N-terminal domains, TIR domain or CC domain, respectively.

Although the functions of each domain in NLR type receptors are not clear so far, there are delicate studies showing the importance of intramolecular and intermolecular interplay among domains in NLR type receptors. A recent study about the composition and structure of ZAR1 (Arabidopsis NLR protein) revealed that activation of ZAR1 forms a wheel-like pentamer, which has a funnel-shaped structure required for immune responsiveness and the CC domain of ZAR1 protein is directly necessary for oligomerization of ZAR1 and shaping a funnel-shaped structure [86]. In other reports, the TIR domain could cleave the metabolic cofactor nicotinamide adenine dinucleotide (NAD^+^) in response to pathogens for turning on downstream signaling [87,88].

### 3.2. Ligand Recognition, Signaling, and Regulation

Ligand recognition by intracellular immune receptors usually induces gene-for-gene resistance (also called effector-triggered immunity or ETI). ETI is typically associated with induced programmed cell death-based defense mechanism called hypersensitive reaction (HR) at locally infected spots [2]. For the resistance response to occur, ligand recognition by the receptor is only the initial step; there are complex downstream signaling steps to regulate immune response.

As a first layer for recognition of effectors or Avr factors secreted by pathogens, some intracellular immune receptors detect their ligands by direct interaction, which turns on defense signaling cascades [85]. For example, tomato I-2, which was introgressed from *Solanum pimpinellifolium*, recognizes Avr2 (also called Secreted in xylem 3, Six3) [89]. Structural-functional analyses of the Avr2 protein reveals that its recognition by the I-2 receptor is not based on surveillance of Avr2 activity in the plant cell, but direct interaction between Avr2 and I-2 [90]. Consistent with these findings, I-2 resistance-breaking Avr2^V41M^, Avr2^R45H^, and Avr2^R46P^ variants, which have mutations on their surface-presented loop, showed normal virulence without recognition by the I-2 receptor [90,91]. This result demonstrated the evolutionary trajectories of the Avr2 effector to avoid I-2 receptor recognition.

There is unique example of direct recognition by intracellular immune receptor, which binds to a conserved PAMP-like region in its target effector. Tomato intracellular receptor Sw-5b recognizes a conserved peptide region NSm^21^ in the viral movement protein NSm from American-type tospoviruses [92]. The NB-ARC-LRR domain of Sw-5b could bind to NSm^21^ peptide directly, which weakens the interaction between NB-ARC and LRR domains thereby activating the downstream immune response [92]. In recent reports, Solanaceae domain of Sw-5b receptor binds to NSm^21^ and this interaction potentiates recognition of NSm^21^ by NB-ARC-LRR region, indicating Sw-5b receptor adopts a two-step recognition mechanism to improve viral effector perception [93].

On the other hand, other intracellular immune receptors indirectly detect their cognate effectors by sensing changes in the status of host proteins targeted by the Avr factors. For example, tobacco immune receptor N could recognize the p50 (50 kDa Helicase of tobacco mosaic virus) by interacting with NRIP1 (N receptor interacting protein 1). The NRIP1-p50 pre-complex allows subsequent interaction between the N receptor and NRIP1-p50 pre-complex for turning on downstream signaling for immunity [94].

Direct or indirect interactions between ligand and receptor induce recognition-mediated changes. These changes include: (1) Intramolecular interactions among domains in receptor, (2) intermolecular interactions among receptor and its interacting proteins, and (3) recruiting or losing nucleotide derivatives to turn on downstream signaling. 

Intramolecular interaction is critical for the function of intracellular immune receptors, as demonstrated in Rx1. Potato immune receptor Rx1 confers high resistance to most Potato Virus X strains and this resistance is initiated by recognition of the viral coat protein (CP) [95,96,97]. Like other receptors, intramolecular cooperation between domains of Rx1 is essential in sensing CP. Mechanistically, recognition of CP disrupts the interaction between its LRR and CC-NB-ARC domains. Recognition-mediated conformational changes and the nucleotide binding state of the protein determines immune activation [98].

Induced intermolecular interactions are also key during invasion pattern recognition and subsequent immune activation. Tomato *Pto,* which is the first isolated R gene and encodes a Serine/Threonine kinase, is involved in the direct recognition of two independent effectors AvrPto and AvrPtoB from *Pseudomonas syringae*, together with the tomato *Prf* NLR gene [99,100,101,102]. Although Pto kinase activity is not essential to bind to their ligand, it is necessary to activate the effector–receptor complex [103,104,105]. In detail, Prf protein could be oligomerized, which induces proximity of two Pto kinase molecules and when effector proteins bind to complexed Pto, this recognition induces trans-phosphorylation on Pto and activates downstream defense signaling [101,106,107]. 

Because the function of immune receptors is directly linked to programmed cell death, it is crucial to maintain NLR proteins folded correctly for keeping their recognition-competent status. As interacting proteins of intracellular immune receptor, HSP90 chaperones and SGT1 (suppressor of the G2 allele of skp1) and RAR1 (required for Mla12 resistance) co-chaperones are involved in NLR-mediated signaling (for extensive review, see Shirasu [108], Kadota et al. [109], and Kadota and Shirasu, [110]).

There are several examples showing functions of HSP90 chaperone and its co-chaperone SGT1 and RAR1 in intracellular immune receptor-mediated resistance in Solanaceous plants. The *Bs2* resistance gene was isolated from pepper plants resistant to strains of *Xanthomonas campestris* pv. *vesicatoria* expressing the effector protein AvrBs2 [111]. Through genetic and molecular biology approaches, the co-chaperone SGT1 was isolated as an interactor of the Bs2 protein in *Nicotiana benthamiana* and necessary for Bs2-AvrBs2-mediated resistance response [112]. In addition, CaSGT1 was identified as a host interactor of AvrBsT, a *X. campestris* pv *vesicatoria* type III effector protein. In pepper (*Capsicum annuum*), CaSGT1 is involved in AvrBsT-triggered hypersensitive reaction [113]. Interestingly, CaSGT1 could bind to CaPIK1, which was also previously reported to be involved in ETI by AvrBsT; CaPIK1-mediated phosphorylation was necessary to promote this response [113].

Recently, UBR7, which is a HECT-type E3 Ubiquitin ligase that recognizes the N-degron in mammals, wasfound to bind to the N immune receptor by proximity labeling [114]. Interestingly, UBR7 induces degradation of the N receptor during normal conditions [114]. When Tobacco mosaic virus (TMV) infects plant cells, p50 inhibits UBR7-mediated degradation of the N receptor by disrupting the interaction between UBR7 and the N receptor [114]. This stabilizes the N receptor only when TMV infection occurs [114].

Although NLR receptors are involved in recognition of their ligands as sensors, some NLRs function as helper receptors. For example, tobacco NRC2 (NLR required for cell death 2) and NRC3 are required for the function of the Prf receptor and NRC4 is required for the function of several NLR receptors including Rpi-blb2, Mi-1.2, and R1 [115]. In addition, NRG1 in *Nicotiana benthamiana* are also necessary for Roq1 (Recognition of XopQ 1) and RPP1-(Recognition of Peronospora parasitica 1) mediated resistance [116]. 

For proper activation of the immune response, subcellular partitioning of intracellular immune receptors is crucial. Through genetic and molecular approaches for dissecting downstream signaling, several plant proteins interacting with Rx1 have been identified. Among them, RanGTPase Activating Protein (RanGAP2), which is involved in nucleocytoplasmic shuttling, was reported to bind Rx1 receptor directly by affinity purification [117,118]. Indeed, RanGAP2 was necessary for Rx1-mediated HR and the RanGAP2-Rx1 interaction retained the Rx1 receptor in the cytoplasm [118,119]. Because hyper-accumulation of nuclear-localized Rx1 receptor by tagging its nuclear localization signal blocks HR and potato virus x (PVX) resistance, this demonstrates that a balanced nucleocytoplasmic partitioning of Rx is necessary for proper regulation of defense signaling [119,120].

As mentioned above, tobacco immune receptor N could recognize the p50-NRIP1 pre-complex. In the absence of infection, NRIP1 protein usually localizes to the chloroplast [94]. After infection, however, the NRIP1 protein could sense and bind to p50; this interaction retains the p50-NRIP1 pre-complex in the cytoplasm, allowing subsequent recognition by N receptor in the cytoplasm [94].

Remarkably, pathogen effector proteins can affect recognition between another effector and its cognate receptor by regulating cell-to-cell movement of the effector. Six5 (Secreted in xylem 5), which is an effector secreted by *Fusarium oxysporum*, is reported to be involved in I-2-mediated resistance [121]. Mechanistically, Six5 localizes and interacts with Avr2 at the plasmodesmata and this interaction allows cell-to-cell movement of Avr2, which potentiates virulence in susceptible plants, but induces I-2-mediated resistance in I-2-containing plants [121]. 

Ultimately, re-organization of gene expression is achieved by recognition of the ligand through receptor-interacting transcription factors. Downstream of the N immune receptor complex, the SPL6 (SQUAMOSA PROMOTER BINDING PROTEIN (SBP)-domain transcription factor) associates with the N immune complex to induce the N-mediated immune response [122]. Recently, the NbGlk1 (Golden2-like transcription factor) was also reported as another interacting protein of the Rx1 receptor [123]. Rx1 could regulate the binding affinity of NbGlk1 to its target DNA sequence, showing a direct mechanistic insight into how NLR activation coordinates immune signaling [123].

## 4. Immune Receptor Crosstalk 

**Figure 1:**
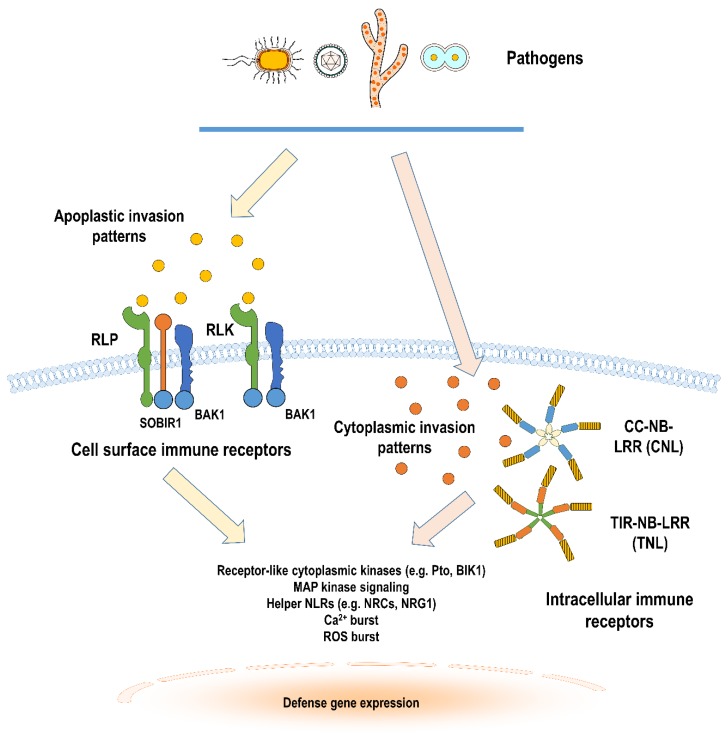
Model of cell surface and intracellular immune receptor-mediated defense signaling in Solanaceous plants. Invasion by pathogens are monitored by plant immune receptors located in the cell surface and cytoplasm. Apoplastic invasion patterns, such as conserved structural epitopes, are recognized by cell surface immune receptors—RLKs (receptor-like kinases), RLPs (receptor-like protein) and their co-receptors. Cytoplasmic invasion patterns such as virulence-promoting effectors are also monitored by NB-LRR (nucleotide-binding-leucine-rich repeat) receptors or NLRs, which could either have coiled coil (CC) or Toll/interleukin-1 receptor (TIR) domains in their N-terminal regions. Downstream of both these types of immune receptors, there are diverse molecular and cellular changes involved in turning on defense signaling. These signaling changes include interaction with various families of receptor kinases localized in the cytoplasm (like Pto and BIK1), phosphorylation cascades of MAP kinases, activation of convergent helper NLR proteins, and production of secondary messengers (like Ca^2+^) or reactive oxygen species (ROS). These ultimately lead to the differential regulation of various transcription factors to carefully tune gene expression according to the appropriate plant host defense output. *(Generated in part using Motifolio Scientific Illustration Toolkits)*.

The distinction between cell surface receptor-mediated PTI and intracellular receptor-mediated ETI has not always been clear cut [157] based on overlapping transcriptome responses [158,159], proteome and phosphoproteome responses [160]. What seemingly sets ETI and PTI is the timing and duration of the immune response. Nonetheless, some exceptions to the supposedly distinct upstream elicitors and downstream defense responses have led to new models of the plant immune system (Figure 1) [161,162].

Recently, the tomato NLR SlNRC4a has been found to directly interact with the cell surface RLP EIX2 and RLK FLS2, providing a direct mechanistic link between the two types of immune receptors [163]. The RLK SERK1 associated with the NLR Mi-1.2 in vivo [164]. NLRs, like tomato NRC1 and tobacco NRC2 and NRC3, have also been shown to be required for immunity directed by the RLP Cf4 [165,166]. This is consistent with studies in Arabidopsis showing direct interaction between NLRs and the PRR FLS2 [167]. Therefore, it comes as no surprise that these two immune receptor types could be directly targeted and suppressed by a common pathogen effector, as shown in potato plants [168].

The transcription factor CAMTA (calmodulin binding transcription activator) has been identified as a convergent master transcription factor by negatively regulating both PTI- and ETI-associated genes in Arabidopsis [169]. Similarly, the tomato CAMTA homologs are involved in negatively regulating resistance to both biotrophic and nectrophic pathogens [170] but it remains to be seen if it directly regulates receptor-mediated immunity genes in tomato plants. 

## 5. Conclusions and Future Perspectives

Excellent and ongoing advances in the study of the plant immune system have increased our understanding of the various immune receptors located at the cell surface and intracellularly. Several studies have provided tremendous insights on the diversity of dangers signal or patterns from the invading pathogen (or from the damaged host itself) recognized by these immune receptors. Although cell surface receptor-triggered and intracellular receptor-triggered immunity have typically been classified as PTI and ETI, respectively, it has been increasingly clear from several studies that these separate distinctions are blurred [157]. One of the most compelling models of the plant innate immune system is describing it as a delicate surveillance system of cell surface and intracellular sensor proteins that detect general invasion or danger signals, whether derived from the attacking pathogens or the damaged hosts [18,161,162].

Inter-species transfer of immune receptor genes for heterologous expression have proven valuable to breeding plant resistance. Arabidopsis RLPs and RLKs, for example, have been transgenically effective in various solanaceous plants [19,171,172,173,174,175,176]. On the other hand, solanaceous immune receptors have been introduced to other species to enhance resistance. This was demonstrated by introducing tobacco FLS2 to Hamlin sweet orange and Carrizo citrange against *Xanthomonas citri* [177]. Tomato Ve1 and Ve2 also conferred resistance to *V. alboatrum* in potato independently [82]. Finally, chimeric receptor hybrids incorporating different domains from different plant clades have also shown novel possibility in producing more resistant plants [178,179,180].

Nonetheless, further research is still required in studying immunity of solanaceous plants, especially since this family represents several agriculturally important crop species and biologically important model species. There remain important knowledge gaps that need to be addressed in the near future. How are RLP/RLK and NLR genes regulated transcriptionally and post-transcriptionally at the mechanistic level, and how do these regulatory processes relate to the well-characterized signal transduction cascade following immune elicitation? How is proper immune homeostasis maintained in the cell through dephosphorylation events, repressor function and autophagy? How are cell surface and intracellular immune receptor proteins trafficked within the cell and what are the crucial molecular components? How similar or different are the downstream mechanisms in plants compared to metazoans? How is the cytoskeleton involved in these defense processes? How does the overlap of downstream molecular components of immune receptors contribute to the growth–defense balance in plants? 

Comprehensive time-scale transcriptomic analyses together with higher-level proteomic, phosphoproteomic and translatomic approaches would allow elucidation of the RLP/RLK and NLR signaling mechanisms at the global level and provide clues on the regulatory hubs that integrate these two tiers of defense responses [160,181,182,183]. The use of mass spectrometry-based interactomic screens targeting various immune receptor protein of interest (under both mock and elicited conditions) could provide clues on downstream interacting proteins (both activator and repressor). Furthermore, newly discovered players in plant immunity are related to diverse processes like autophagy, vesicular trafficking and cytoskeleton [184,185,186]. Characterization of homologous solanaceous plant genes through CRISPR/Cas9 genome editing would expand our knowledge of genes that perturbs host resistance responses [187]. Downstream detailed phenotyping of these genome-edited plants in terms of biomass, photosynthetic rates and respiratory efficiency would allow us insights into the host’s growth–defense balance.

Other interesting biological questions extend the dimension beyond binary host–pathogen interaction. How do various environmental factors affect immune receptor-mediated defense outputs and signaling events? How does the plant-microbiome crosstalk synergistically or antagonistically interact with immune receptor function and signaling? How can we harness natural variation in the plant immune system for breeding crop resistance? 

It would be great to mechanistically investigate various environmental regimes and their impact on plant immune responses, with particular attention paid to specific molecular outputs related to surface and/or intracellular receptor signaling. For example, infection with the tomato leaf curl virus modulates molecular chaperones crucial for the heat tolerance response [188]. Further deciphering the mechanistic link of changing environmental factors to immune receptor signaling would prove important in identifying other vulnerable signaling regulators. Apart from looking at abiotic environmental parameters, examining the biotic contributions of the microbiome could inform future management and mitigation strategies. This would include assessing microbiota assembly in isogenic lines that differ in their resistance or testing differing synthetic communities [189,190]. 

Finally, exploring beyond model solanaceous plants would open larger sources of continuously evolving immune receptor proteins. It has been shown that diverse tomato accessions were able to mount differential immune responses that relied on pattern-triggered immunity based on reporter genes as defense outputs. However, the receptors and molecular mechanisms still need to be identified and elucidated [191]. Together, these questions make the study of plant immunology quite exciting and rewarding in *Solanaceae* and beyond.

## Figures and Tables

**Table 1:** Known cell surface immune receptors proteins in plant species of the *Solanaceae* family.

Receptor	Type	Species	Ligand	Ligand Source	Reference
CSPR	RLK	*Nicotiana benthamiana*	csp22 (cold shock protein)	Pathogen (various bacterial species)	Saur et al. [56]
CORE	RLK	*Solanum* *lycopersicum*	csp22 (cold shock protein)	Pathogen (various bacterial species)	Wang et al. [16]
FLS2	RLK	*Solanum* *lycopersicum*	flg22, flg15 (flagellin)	Pathogen (various bacterial species)	Robatzek et al. [7]
FLS3	RLK	*Solanum* *lycopersicum*	flgII-28 (flagellin)	Pathogen (various bacterial species)	Hind et al. [6]
I-3	RLK	*Solanum* *lycopersicum*	Avr3	Pathogen (*Fusarium oxysporum*)	Catanzariti et al. [57]
LRPK1	RLK	*Solanum* *tuberosum*	?	Pathogen (*Phytophthora infestans*)	Wang et al. [58]
Nt-Sd-RLK	RLK	*Nicotiana tabacum*	LPS (lipopolysaccharide)?	Pathogen (bacteria)	Sanabria et al. [10]
PEPR1	RLK	*Solanum* *lycopersicum*	SlPep6	Host	Lori et al. [59]
PSKR1	RLK	*Solanum* *lycopersicum*	PSK (phytosulfokine)	Host	Zhang et al. [60]
PSKR2	RLK	*Solanum* *lycopersicum*	PSK (phytosulfokine)	Host	Zhang et al. [60]
Rmprp-1	RLK	*Capsicum annuum*	?	Pathogen (*Myzus persicae*)	Sun et al. [61]
SlLYK1	RLK	*Solanum* *lycopersicum*	Chitin?	?	Liao et al. [9]
SR160	RLK	*Solanum* *lycopersicum*	?	?	Scheer and Ryan [62]
SYR1	RLK	*Solanum* *lycopersicum*	systemin	Host	Wang et al [8]
SYR2	RLK	*Solanum* *lycopersicum*	systemin	Host	Wang et al [8]
Cf-2	RLP	*Solanum* *lycopersicum*	Avr2	Pathogen (*Cladosporium fulvum*)	Dixon et al. [63]; Jones et al. [64]; Thomas et al. [65]
Cf-4	RLP	*Solanum* *lycopersicum*	Avr4	Pathogen (*C. fulvum*)	Thomas et al. [66]; Jones et al. [64]; Thomas et al. [65]
Cf-4A (Hcr9-4E)	RLP	*Solanum* *lycopersicum*	Avr4E	Pathogen (*C. fulvum*)	Takken et al. [67]; Takken et al., 1998 [68]
Cf-5	RLP	*Solanum* *lycopersicum*	Avr5	Pathogen (*C. fulvum*)	Dixon et al. [69]; Jones et al. [64]; Thomas et al. [65]
Cf-6	RLP	*Solanum* *lycopersicum*	?	Pathogen (*C. fulvum*)	Grushtskaia et al. [70]
Cf-9	RLP	*Solanum* *lycopersicum*	Avr9	Pathogen (*C. fulvum*)	Jones et al., 1994 [71]; Hammond-Kossack et al. [72]; Hammond-Kossack et al. [73]; van der Hoorn et al. [74]
Cf-9B (Hcr9-9B)	RLP	*Solanum* *lycopersicum*	?	Pathogen (*C. fulvum*)	Parniske et al. [75]
Cf-ECP2	RLP	*Solanum* *lycopersicum*	ECP2	Pathogen (*C. fulvum*)	Laugé et al. [76]
CuRe1	RLP	*Solanum* *lycopersicum*	?	Parasitic plant (*Cuscuta reflexa*)	Hegenauer et al. [21]
EIX1	RLP	*Solanum* *lycopersicum*	Ethylene-inducing xylanase (EIX)	Pathogen (*Trichoderma* spp.)	Ron and Avni [77]; Bar et al. [78]
EIX2	RLP	*Solanum* *lycopersicum*	Ethylene-inducing xylanase (EIX)	Pathogen (*Trichoderma* spp.)	Ron and Avni [77]
ELR	RLP	*Solanum* *tuberosum*	INF1 elicitin	Pathogen (*P. infestans*)	Du et al. [19]
I	RLP	*Solanum* *lycopersicum*	Avr1	Pathogen (*F. oxysporum*)	Catanzariti et al. [79]
I-7	RLP	*Solanum* *lycopersicum*	?	Pathogen (*F. oxysporum*)	Gonzalez-Cendales et al. [80]
RXEG1	RLP	*Nicotiana benthamiana*	XEG1 (glycoside hydrolase 12 protein)	Pathogen (*P. sojae*)	Wang et al. [81]
Ve1	RLP	*Solanum* *lycopersicum*	Ave1	Pathogen (*Verticillium* *dahliae, V. albo-atrum*)	Kawchuk et al. [82]; Fradin et al. [83]; Castroverde et al. [37]
Ve2	RLP	*Solanum* *lycopersicum*	?	Pathogen (*V. dahliae, V. albo-atrum*)	Kawchuk et al. [82]; Nazar et al. [84]

**Table 2:** Known intracellular immune receptors proteins in plant species of the *Solanaceae* family.

Receptor	Type	Species	Ligand	Ligand Source	Reference
Bs2	CNL	*Capsicum annuum*	AvrBs2	*Xanthomonas campestris*	Swords et al. [124]; Tai et al. [125]
Gpa2 (Rxh1)	CNL	*Solanum* *tuberosum*	RBP-1	*Globodera pallida*	van der Vossen et al. [126]
Hero	CNL	*Solanum* *lycopersicum*		*Globodera rostochiensis, G. pallida*	Ernst et al. [127]
I-2	CNL	*Solanum* *lycopersicum*	Avr2	*Fusarium oxysporum*	Ori et al. [128] Simons et al. [129]
Mi-1.2	CNL	*Solanum* *lycopersicum*		*Meloidogyne incognita, M. arenaria,* *M. javanica, Bemisia tabacci*	Milligan et al. [130] Vos et al. [131]
Prf	CNL	*Solanum* *lycopersicum*	AvrPto, AvrPtoB	*Pseudomonas syringae*	Salmeron et al. [101]; Abramovitch et al. [132]; Ronald et al. [133]
R1	CNL	*Solanum* *tuberosum*	Avr1	*Phytophthora infestans*	Ballvora et al. [134]; van der Lee et al. [135]
R3a	CNL	*Solanum* *tuberosum*	Avr3a	*Phytophthora infestans*	Huang et al. [136]; Armstrong et al. [137]
R8	CNL	*Solanum tuberosum*	Avr8	*Phytophthora infestans*	Vossen et al. [138]
Rpi-blb1 (RB)	CNL	*Solanum* *bulbocastanum*	Avrblb1	*Phytophthora infestans*	Song et al. [139]; van der Vossen et al. [140]; Oh et al. [141]
Rpi-blb2	CNL	*Solanum* *bulbocastanum*	Avrblb2	*Phytophthora infestans*	van der Vossen et al. [142]; Oh et al. [141]
Rx1	CNL	*Solanum* *tuberosum*	CP	Potato virus x	Bendahmane et al. [95]
Rx2	CNL	*Solanum acaule*	CP	Potato virus x	Bendahmane et al. [143]
Sw-5	CNL	*Solanum* *lycopersicum*		Tospovirus	Brommonschenkel et al. [144]
Sw-5b	CNL	*Solanum tuberosum*	NSm (viral movement protein)	Tospovirus	Hallwass et al. [145]; Zhu et al. [92]
Tm-2	CNL	*Solanum* *lycopersicum*	MP	Tomato mosaic virus	Calder et al. [146]; Lanfermeijer et al. [147]
Tm-2-2	CNL	*Solanum* *lycopersicum*	MP	Tomato mosaic virus	Calder et al. [146]; Lanfermeijer et al. [148]
Rpa1	CNL	*Nicotiana tabacum*	AvrRpm1_psa_	*Pseudomonas syringae pv. actinidiae*	Yoon and Rikkerink [149]
Bs4	TNL	*Solanum* *lycopersicum*	AvrBs4, Hax4	*Xanthomonas campestris*	Bonas et al. [150]; Kay et al. [151]; Schornack et al. [152]
Gro1-4	TNL	*Solanum* *tuberosum*		*Globodera rostochiensis*	Paal et al. [153]
N	TNL	*Nicotiana tabacum*	Helicase	Tobacco mosaic virus	Erickson et al. [154]; Whitham et al. [155]
Roq1	TNL	*Nicotiana benthamiana*	XopQ HopQ1	*Xanthomonas campestris* *Pseudomonas syringae*	Schultink et al. [156]
